# A Hypoxia-Associated Prognostic Gene Signature Risk Model and Prognosis Predictors in Gliomas

**DOI:** 10.3389/fonc.2021.726794

**Published:** 2021-11-12

**Authors:** Feng Gao, Zhengzheng Wang, Jiajie Gu, Xiaojia Zhang, Huixiao Wang

**Affiliations:** Department of Neurosurgery, The Affiliated People’s Hospital of Ningbo University, Ningbo City, China

**Keywords:** hypoxia, glioma, risk prediction model, Cox regression, ubiquitin–proteasome system

## Abstract

Most solid tumours are hypoxic. Tumour cell proliferation and metabolism accelerate oxygen consumption. The low oxygen supply due to vascular abnormalisation and the high oxygen demand of tumour cells give rise to an imbalance, resulting in tumour hypoxia. Hypoxia alters cellular behaviour and is associated with extracellular matrix remodelling, enhanced tumour migration, and metastatic behaviour. In light of the foregoing, more research on the progressive and prognostic impacts of hypoxia on gliomas are crucial. In this study, we analysed the expression levels of 75 hypoxia-related genes in gliomas and found that a total of 26 genes were differentially expressed in The Cancer Genome Atlas (TCGA) database samples. We also constructed protein–protein interaction networks using the STRING database and Cytoscape. We obtained a total of 10 Hub genes using the MCC algorithm screening in the cytoHubba plugin. A prognostic risk model with seven gene signatures (PSMB6, PSMD9, UBB, PSMD12, PSMB10, PSMA5, and PSMD14) was constructed based on the 10 Hub genes using LASSO–Cox regression analysis. The model was verified to be highly accurate using subject work characteristic curves. The seven-gene signatures were then analysed by univariate and multivariate Cox. Notably, PSMB10, PSMD12, UBB, PSMA5, and PSMB6 were found to be independent prognostic predictive markers for glioma. In addition, PSMB6, PSMA5, UBB, and PSMD12 were lowly expressed, while PSMB10 was highly expressed, in the TCGA and GTEx integrated glioma samples and normal samples, which were verified through protein expression levels in the Human Protein Atlas database. This study found the prognostic predictive values of the hypoxia-related genes PSMB10, PSMD12, UBB, PSMA5, and PSMB6 for glioma and provided ideas and entry points for the progress of hypoxia-related glioma.

## Introduction

There are more than 100 histological subtypes of primary brain and central nervous system (CNS) tumours (1). Gliomas account for 24% of all primary brain and CNS tumour types, including low-grade gliomas (LGG) and glioblastoma multiforme (GBM), and are the most common and lethal type of primary malignancies of the CNS (2). Currently, although surgical resection, chemotherapy, and radiotherapy are considered clinically standard treatments for gliomas (3), treatment efficacy is very limited, with no cure for gliomas and very poor prognosis for patients (4, 5). In addition, most glioma patients are prone to drug resistance and relapse during treatment (6, 7). Therefore, the search for new molecular therapeutic targets and prognostic predictive markers is important to predict treatment response and clinical outcome in glioma.

Tumour hypoxia is a condition in which tumour cells are deprived of oxygen (8). During the growth of malignant tumours, the tumour cells grow faster than the blood vessels; therefore, the blood supply cannot keep up with the demand that matches the tumour size, leaving parts of the tumour with significantly lower oxygen concentrations than healthy tissues, resulting in a hypoxic microenvironment (9, 10). The hypoxic tumour microenvironment is widely recognised as an independent prognostic indicator that is commonly associated with low survival rates in various cancer types, including breast and lung cancers. In gliomas, hypoxia is a driver of the malignant phenotype of the glioma class (11). Tumour hypoxia is associated with antiapoptosis, recurrence, chemo- and radiotherapy resistance, invasive potential, and reduced patient survival (12).

Cancer cells have multiple mechanisms for evading radiotherapy-induced cell death. Among them, the development of tumour hypoxia and its associated metabolic pathways is one of the most important contributors to clinical radioresistance (13). This is attributed to the fact that hypoxic tumours require approximately three times the normal radiation dose to achieve the desired cell death (14). This likewise suggests that a tumour hypoxia greatly reduces the efficacy of conventional anticancer approaches. Previous studies have shown that T cells and natural killer cells present an incompetent or depleted state in a hypoxic microenvironment, which results in dysfunction (15, 16). Currently, the predictive biomarkers for immunotherapy mainly include programmed death-ligand 1 (PD-L1), microsatellite instability/defective mismatch repair (MSI/dMMR), and tumour mutational load (TMB) but often ignore the hypoxic tumour microenvironment as a prerequisite (17). Recent studies (18) have constructed and validated a hypoxia risk model that serves as an independent prognostic indicator for glioma, reflecting the overall strength of the immune response in a glioma microenvironment. However, it is still difficult to determine the hypoxic status of tumours due to the diversity of oxygen levels in different tissues. Under hypoxic conditions, tumour cells can adapt to the microenvironment where they grow by altering the expression of endogenous enriched genes; these gene expression profiles can reflect the hypoxic status (19, 20). Therefore, exploring the exact or relevant mechanisms of hypoxia in tumour development is expected to provide new targets and indicators for the treatment and prognosis detection of gliomas.

In this study, we analysed the expression and correlation of 75 hypoxia-related genes in gliomas and thereafter constructed a highly accurate prognostic risk prediction model consisting of seven gene signatures. PSMB10, PSMD12, UBB, PSMA5, and PSMB6 were found to be independent predictors of glioma prognosis.

## Method

### Data Sources

The data of 663 glioma (GBM + LGG) samples, and mRNA expression data, were downloaded from The Cancer Genome Atlas (TCGA, https://portal.gdc.cancer.gov/) website, while the mRNA expression data of 2,642 cases of normal tissues were downloaded from the Genotype-Tissue Expression (GTEx, https://gtexportal.org/) website. The 75 hypoxia-associated genes were cited in Wei et al. (21).

### Selection and Processing of Hypoxia-Associated Genes

The collected data were normalised, and 2,642 normal lung tissues from GTEx were added to the glioma TCGA dataset. The R package (v4.0.3) was used to analyse the differences in the 75 hypoxia-associated gene expressions. Correlations between quantitative variables without a normal distribution were described using Spearman’s correlation analysis. p < 0.05 was considered statistically significant.

### Seventy-Five Hypoxia-Associated Gene Subgroup Types

Consistency analysis was performed using the R package ConsensusClusterPlus (v1.54.0) with a maximum number of clusters of six and 100 replicates to extract 80% of the total sample, clustering = “hc”, innerLinkage = ‘ward.D2’. The clustering heatmaps were all analysed using the R software package pheatmap (v1.0.12). The gene expression heatmaps were retained for genes with variances above 0.1.

### Protein–Protein Interaction Network Construction and Hub Gene Screening

The STRING database (https://string-db.org/) was used to identify known and predicted PPIs. Seventy-five hypoxia-associated genes were analysed, and PPI networks were constructed using STRING. The top 10 Hub genes in the PPI networks were further screened using cytoHubba in Cytoscape (v3.8.2) software.

### Kaplan–Meier Survival Analysis

Survival analysis was performed using Survival in the R package. The p-values and hazard ratios (HR) with 95% confidence intervals (CI) in the Kaplan–Meier curves were derived through log-rank test and univariate Cox proportional hazards regression.

### LASSO Model Construction

The LASSO regression algorithm was used for feature selection, and 10-fold cross-validation was used to determine the parameters needed to obtain a suitable model. The genes obtained from LASSO regression were then subjected to multifactor Cox regression analysis, and the multifactor regression coefficient of each gene was calculated to construct the risk score equation. The patients were divided into high- and low-risk groups according to the median risk score values. The Kaplan–Meier survival curve analysis was used to compare the overall survival times of the two groups, and the predictive value of the genetic markers was evaluated through time-related receiver operating characteristic (ROC).

### Univariate and Multivariate Cox Regression Analysis

Cox regression analysis was performed using the Survival package, and forest plots were plotted using the forestplot package to obtain the p-value, HR, and 95% CI for each variable. Based on the results of the multivariate Cox proportional risk analysis, column line plots were constructed using the RMS package to predict the 1-, 3-, and 5-year survival rates.

### Protein Expression Validation

Immunohistochemical staining maps of the gene expression in both glioma tissues and normal tissues were downloaded from the Human Protein Atlas (HPA) database.

### Gene Set Enrichment Analysis

Samples were divided into two groups of high and low expression according to the median value of gene expression, and the enrichment of Kyoto Encyclopedia of Genes and Genomes (KEGG) and HALLMARK pathways in the high and low expression groups were analysed using gene set enrichment analysis (GSEA).

## Results

### Expression and Correlation of Hypoxia-Associated Genes in Gliomas

**Figure 1:**
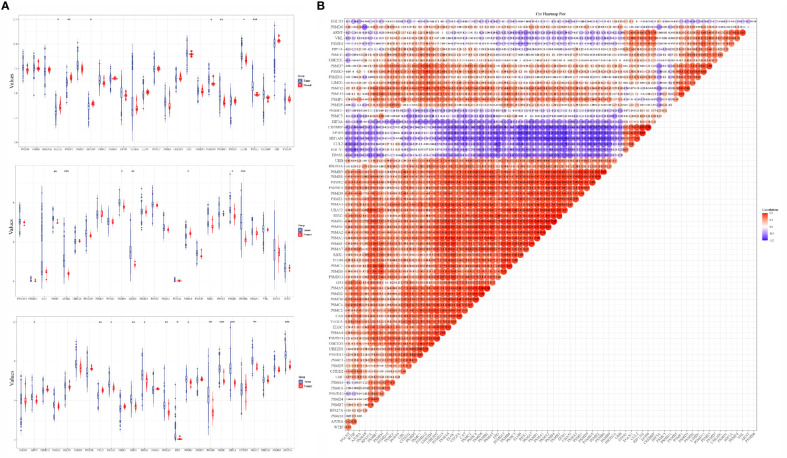
Expression levels and correlations of hypoxia-related genes in the TCGA database. **(A)** Expression levels of 75 hypoxia-related genes in the TCGA database and **(B)** correlation of expression levels of 75 hypoxia-related genes in the TCGA database glioma samples. *p < 0.05, **p < 0.01, ***p < 0.001.

The analysis results of the expression levels of 75 hypoxia-related genes in 663 glioma samples and five paraneoplastic tissue samples from the TCGA database showed that EGLN2, PSMD1, HIF1AN, PSMD10, PSMB10, ELOB, PSME2, PSMF1, AJUBA, PSMB4, LIMD1, PSMC6, PSMB1, PSMB8, ARNT, GUL2, PSMA3, SEM1, EPAS1, PSMA2, EPO, PSME3, PSMB9, HIF1A, UBA52, and RPS27A were significantly differentially expressed in cancer and paraneoplastic tissues (**Figure 1A**). The analysis further revealed that most of the 75 hypoxia-related genes were positively correlated. Among them, PSMB3 was the most significantly correlated with PSMB6 (**Figure 1B**). This suggests that when PSMB3 is upregulated, the PSMB6 gene is most likely to be upregulated.

### Consensus Clustering of Hypoxia-Associated Genes

**Figure 2:**
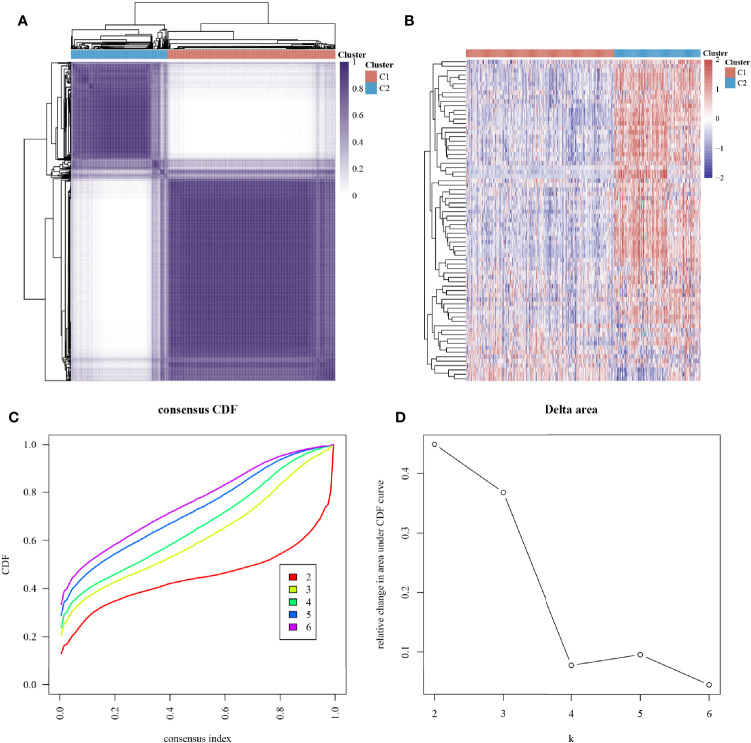
Subtype grouping of 75 hypoxia-associated genes in the TCGA database glioma samples. **(A)** Consensus clustering matrix at K = 2, **(B)** ConsensusClusterPlus consistency clustering result heatmap at K = 2, **(C)** CDF curve at K = 2–6, and **(D)** CDF Delta area curve at K = 2–6.

The ConsensusClusterPlus package was used to classify the subgroups of the 663 glioma samples, which were identified as having the best cluster stability from K = 2 to 6 when K = 2 (**Figure 2**). The 663 glioma patients were subsequently classified into two subgroups, namely, cluster 1 (C1, n = 421) and cluster 2 (C2, n = 242), based on the expression levels of the hypoxia-related genes.

### Expression of Hypoxia-Associated Genes in Different Subgroups and Clinical Characteristics of Glioma Patients

**Figure 3:**
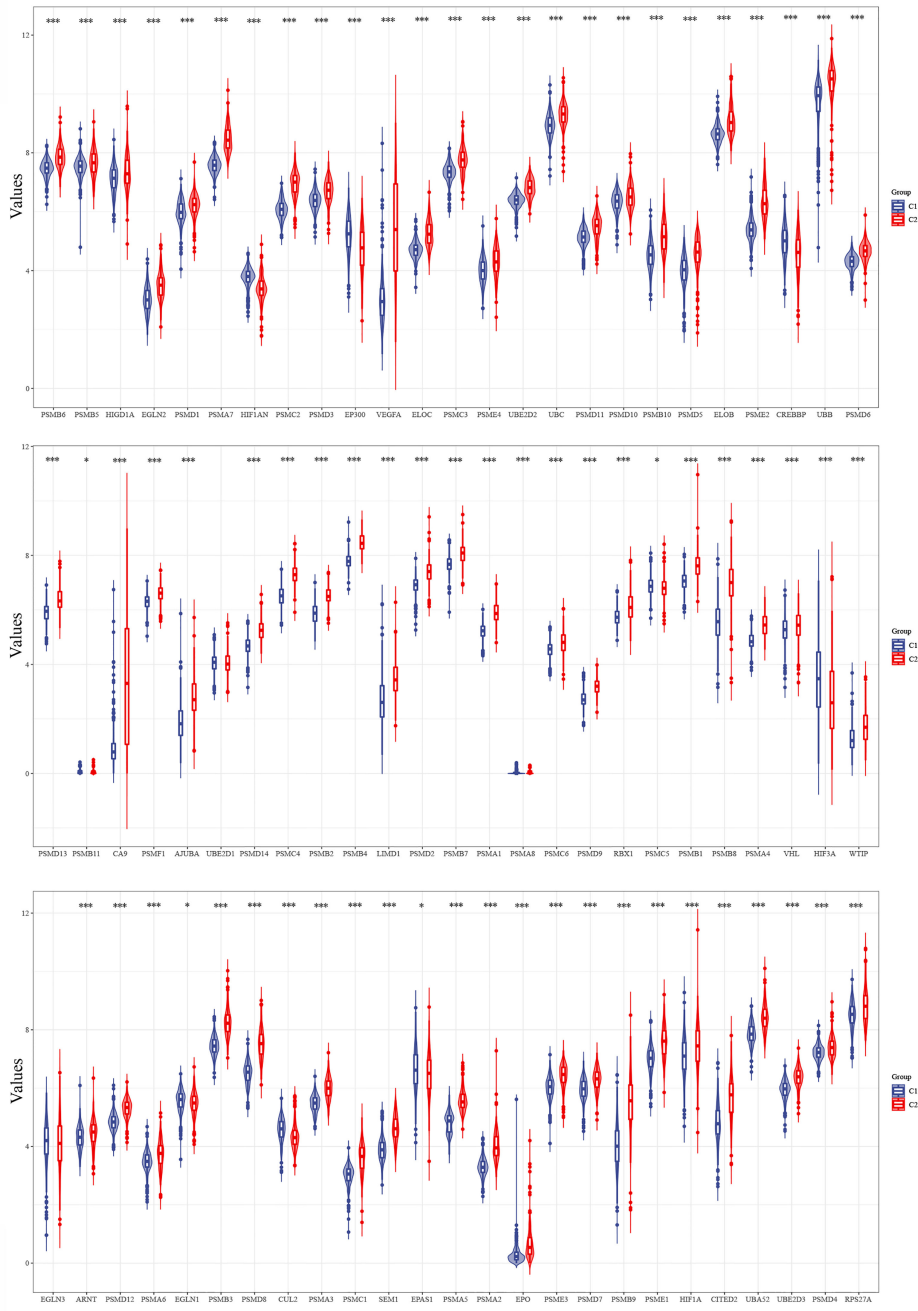
Differences in the expression levels of 75 hypoxia-related genes in the two subgroups (*p < 0.05, ***p < 0.001).

The expression levels of 75 hypoxia-associated genes were observed in the two subgroups (**Figure 3**). The results showed that except for UBE2D1 and EGLN3, the differences in the expression levels of the remaining 72 hypoxia-related genes in the two subgroups were statistically significant (p < 0.05). The distribution of clinical data and the survival of the samples in the two subgroups are shown in **Table 1**, thereby underscoring the significant differences (p < 0.05) between the two groups in terms of tumour histological grade, and the need (or not) for radiotherapy and chemotherapy (**Supplementary Figure S1**).

**Table 1:** Distribution of clinical data of the samples in the two subgroups.

	Characteristic	C1 (n = 421)	C2 (n = 242)
Status	Alive	338	78
	Dead	83	164
Age	Mean (SD)	42 (13.5)	55.1 (14.4)
	Median[MIN,MAX]	39 [14,87]	57 [21,89]
Gender	Female	182	100
	Male	239	142
Race	American Indian	1	0
	Asian	6	7
	Black	16	15
	White	389	218
Grade*	Discrepancy	1	0
	G2	236	12
	G3	179	82
New tumour event type	Progression	2	62
	Recurrence	–	16
Radiation therapy*	Non-radiation	109	11
	Radiation	104	39
History of neoadjuvant therapy	Yes	3	
	No	418	242
Therapy type*	Ancillary : Chemotherapy:Targeted Molecular Therapy	1	
	Chemotherapy	175	107
	Chemotherapy:	5	2
	Chemotherapy : Targeted Molecular Therapy	1	–
	Chemotherapy : Hormone Therapy	1	15
	Chemotherapy : Hormone Therapy : Immunotherapy:	1	–
	Chemotherapy : Hormone Therapy : Other (specify in notes)	3	–
	Chemotherapy : Immunotherapy	8	5
	Chemotherapy : Other (specify in notes)	3	1
	Chemotherapy : Targeted Molecular Therapy	17	36
	Immunotherapy	2	–
	Chemotherapy : Hormone Therapy : Other (specify in notes):Targeted Molecular Therapy	–	1
	Chemotherapy : Hormone Therapy : Targeted Molecular therapy	–	1
	Chemotherapy : Immunotherapy:Targeted Molecular Therapy	–	2
	Hormone Therapy	–	6
	Hormone Therapy : Targeted Molecular Therapy	–	1

*p < 0.05.

### PPI Network Construction and Hub Gene Identification

**Figure 4:**
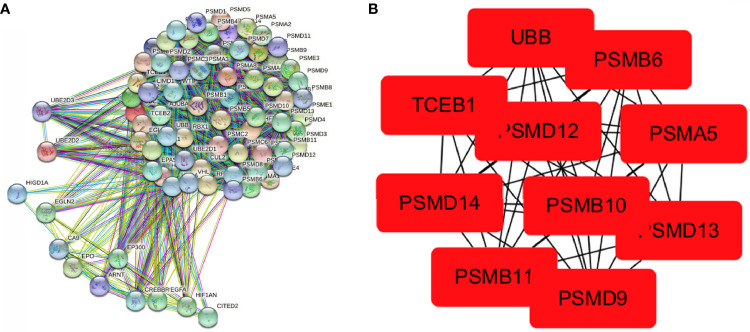
Visualisation of the protein–protein interaction network and Hub genes. **(A)** PPI network map of 78 hypoxia-associated genes and **(B)** screening of Hub genes using the MCC algorithm.

A PPI network of 75 hypoxia-related genes, including 75 nodes and 2,110 edges, was constructed using the STRING database (**Figure 4A**). The top 10 Hub genes with the highest linkage degrees were then obtained using the MCC algorithm of the cytoHubba plugin in the Cytoscape software, namely, PSMB6, PSMD9, UBB, PSMD12, PSMB10, PSMB11, PSMD13, PSMA5, PSMD14, and TCEB1 (**Figure 4B**).

### LASSO Prognostic Model Construction

**Figure 5:**
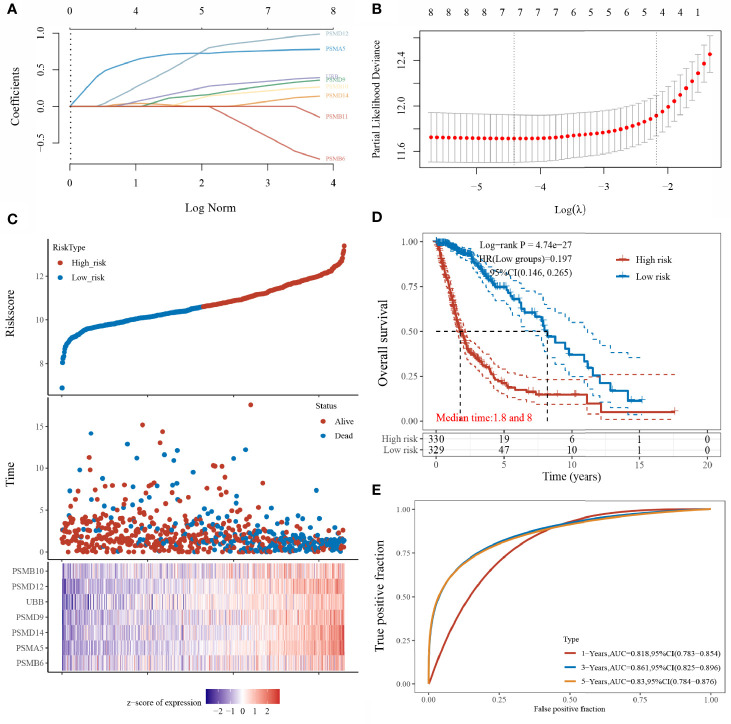
Construction of the LASSO prognostic model. **(A)** Coefficients of selected features shown by lambda parameters; **(B)** partial likelihood deviation plotted against log(λ) using LASSO-Cox regression model; **(C)** risk score and survival time with survival status profile and expression levels of the seven gene signatures; **(D)** Kaplan–Meier curves for patients in the high- and low-risk groups; and **(E)** ROC curve validation (LASSO model for 1-, 3-, and 5-year prediction accuracies).

The prognostic features were constructed using the LASSO-Cox regression model to analyse the expression levels of the Hub genes. A prediction model with seven gene signatures (**Figures 5A, B**) was constructed according to the minimum criterion (Lambda.min = 0.0121), selecting PSMB6, PSMD9, UBB, PSMD12, PSMB10, PSMA5, and PSMD14, whose predicted risk scores consisted mainly of the following:


$$\begin{array}{c} \mathrm{Riskscore}=(-0.5071)*\text{PSMB}6+(0.3068)*\text{PSMD}9\\ +(0.3587)*\text{UBB}+(0.9338)*\text{PSMD}12\\ +(0.2287)*\text{PSMB}10+(0.7667)*\text{PSMA}5\\ +(0.0892)*\text{PSMD}14 \end{array}$$

The sample was divided into high- and low-risk groups (**Figure 5C**) according to the risk score ranking, with the median risk score as the threshold. The results of the Kaplan–Meier analysis showed that patients in the high-risk group had significantly worse prognoses than those in the low-risk group (**Figure 5D**). In addition, the sensitivity and specificity of the model for predicting the patients’ OS periods were verified by applying the ROC curves. We found that the present risk model predicted AUC values of 0.818, 0.861, and 0.830 for the 1-, 3-, and 5-year prognosis, respectively. This indicates that the model has high accuracy in predicting the prognostic survival of glioma patients (**Figure 5E**).

### Univariate and Multivariate Cox Regression Analysis

**Figure 6:**
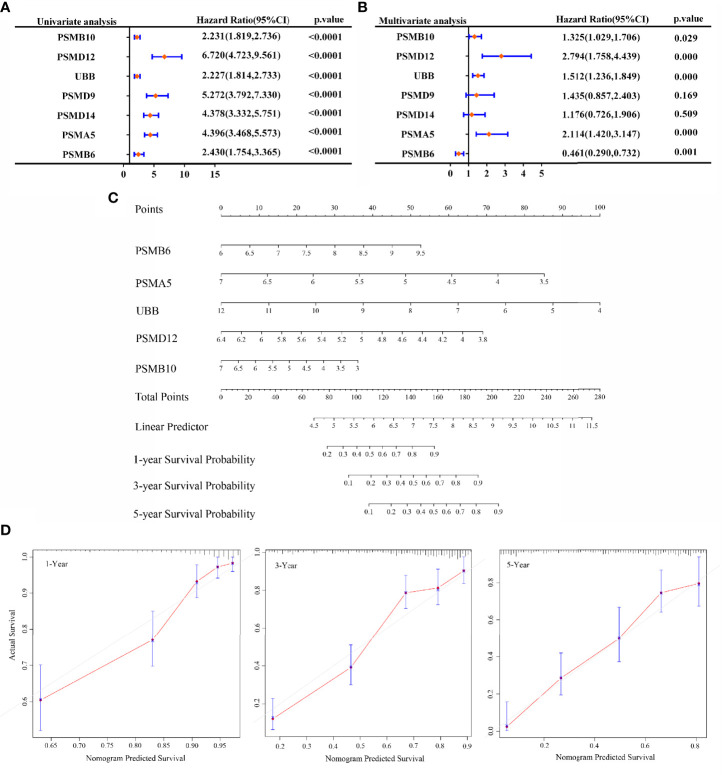
Prognosis prediction by Cox analysis of seven gene signatures. **(A)** p-value, risk factor HR, and confidence interval for single-factor Cox analysis of seven gene signature expressions and clinical characteristics. **(B)** p-value, risk factor HR, and confidence interval for multifactor Cox analysis of seven gene signature expressions and clinical characteristics. **(C)** Column line graphs predicting overall survival at 1, 3, and 5 years for glioma patients. **(D)** Calibration curves of the overall survival column line graph model.

In this study, our objective was to analyse whether SMB6, PSMD9, UBB, PSMD12, PSMB10, PSMA5, and PSMD14 are independent prognostic factors for glioma. Univariate and multifactorial COX regression analyses were used to determine that PSMB10, PSMD12, UBB, PSMA5, and PSMB6 may be independent prognostic factors for gliomas (**Figures 6A, B** **)**. Next, we generated a nomogram using COX regression to construct a model for predicting the overall survival at 1, 3, and 5 years (**Figure 6C**). The calibration results showed that the 1-, 3-, and 5-year overall survival models had good predictive properties compared with the ideal model (**Figure 6D**).

### Expressions and Protein Assays of PSMB10, PSMD12, UBB, PSMA5, and PSMB6 in Gliomas

**Figure 7:**
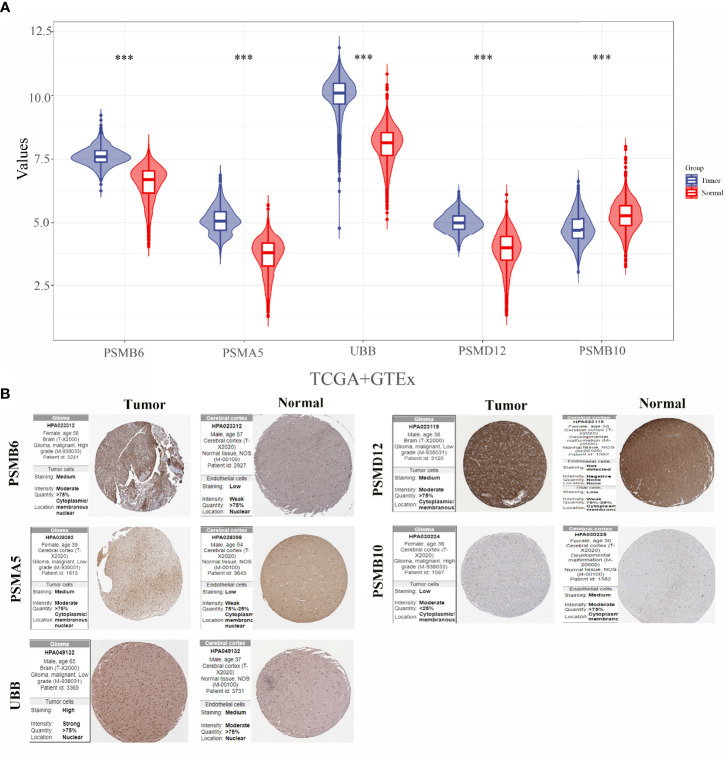
Expressions and protein validations of PSMB10, PSMD12, UBB, PSMA5, and PSMB6 in gliomas. **(A)** Expression levels of five genes in glioma samples from the TCGA and GTEx databases and **(B)** protein expression levels of five genes in glioma tissues and normal brain tissues from the HPA database. ***p < 0.001.

The expression levels of PSMB10, PSMD12, UBB, PSMA5, and PSMB6 were analysed by integrating 663 glioma cancer tissue samples and 5 paraneoplastic tissue samples from the TCGA database and 2,642 normal tissue samples from the GTEx database. The results showed that PSMB6, PSMA5, UBB, and PSMD12 were significantly downregulated, and PSMB10 was significantly upregulated in gliomas (**Figure 7A**). The protein expressions of the five genes in the glioma cancer tissues and normal tissues were verified using the HPA online database (**Figure 7B**). The results showed that PSMB6, PSMA5, UBB, and PSMD12 were highly expressed in the glioma tissues, while PSMB10 was lowly expressed in the glioma tissues compared with the normal tissues.

### Gene Set Enrichment Analysis

**Figure 8:**
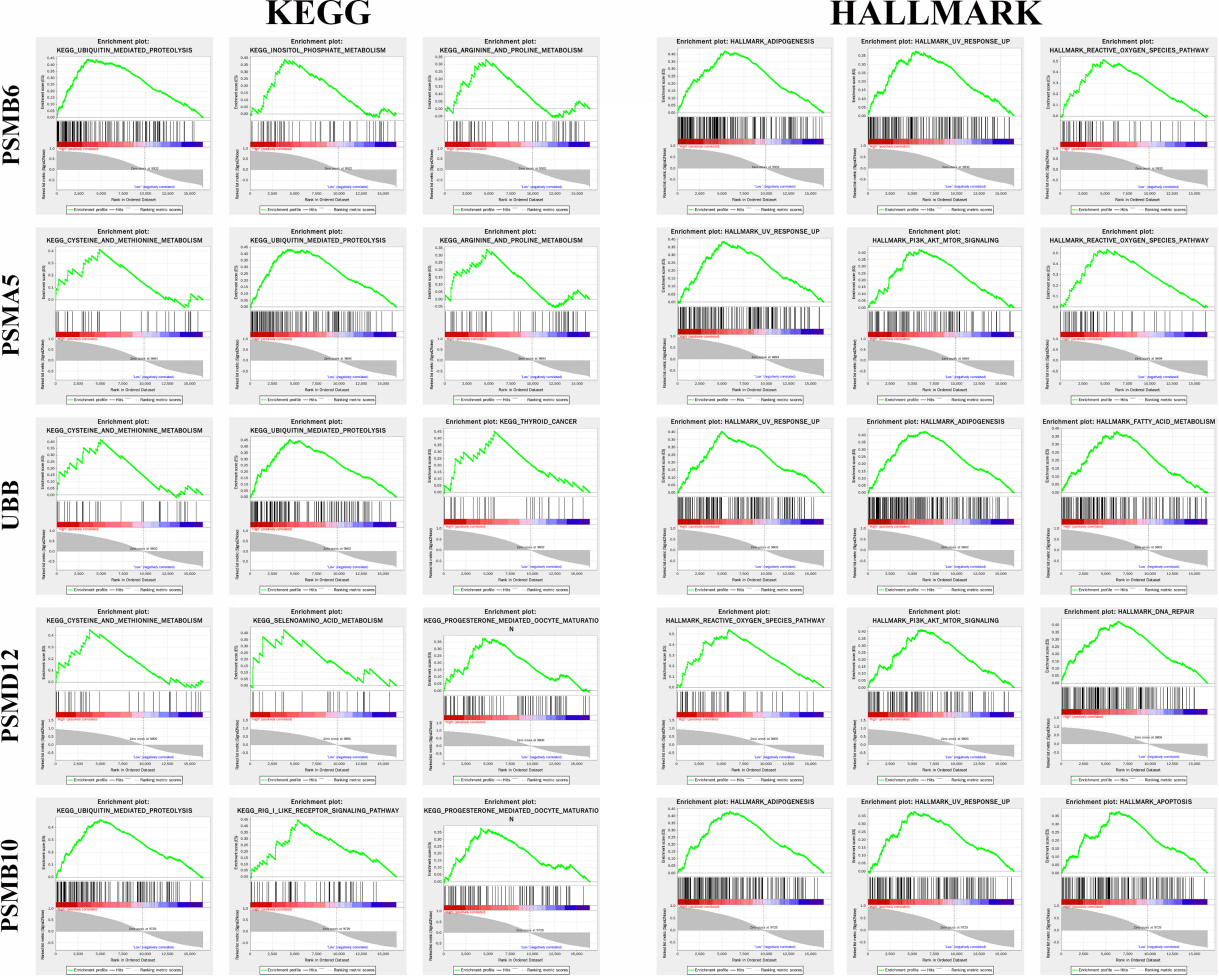
Gene set enrichment analysis of the genes in gliomas.

**Figure 8** shows the top 3 most abundant signalling pathways or biological processes, respectively, ranked according to normalized enrichment score (NES) values of PSMB10, PSMD12, UBB, PSMA5, and PSMB6 in gliomas. As the results showed, high PSMB6, PSMA5, UBB, and PSMB10 expressions were all enriched in ubiquitin-mediated proteolysis and UV response pathway. PSMD12 was enriched in cysteine and methionine metabolism and reactive oxygen species pathway.

## Discussion

Hypoxia is one of the main features of a glioma (22). The presence of hypoxia in human gliomas has been experimentally demonstrated in previous studies (23). Furthermore, the close correlation between hypoxia and radioresistance in gliomas has been reported in numerous studies (24, 25). Radiation therapy targets rapidly proliferating tumour cells mainly by increasing reactive oxygen species (ROS)-induced oxidative stress. Reactive oxygen molecules include superoxide radicals and hydroxyl radicals. ROS break chemical bonds, activate cascade reactions generated by DNA damage, and ultimately lead to cell death. In this process, oxygen is the key to mitigating ROS-induced DNA damage, which is the fundamental mechanism of radiation for cancer therapy (26). Tumour hypoxic zones recruit some immunosuppressive cells, such as bone marrow-derived suppressor cells (MDSCs), tumour-associated macrophages (TAMs) and Tregs, and thereafter inhibit the activation of CD8+ T and CD4+ T cells (27, 28). Under hypoxic conditions, cancer cells upregulate the key metabolic enzymes that help them adapt to the demand for nutrients and the changes in redox status (29). Therefore, understanding the molecular mechanisms underpinning the effects of hypoxia on tumour treatment is crucial to improve the effectiveness of tumour therapy.

In the current study, we applied consistent clustering, a method that provides quantitative evidence for determining the number and membership of possible clusters in a dataset, to hypoxia-related genes. We divided 663 glioma samples into two subgroups by consistency clustering, and our analysis showed significant correlations between the subgroup level and the tumour histological grade, radiotherapy or lack thereof, and chemotherapy modality. We also constructed a glioma prognostic prediction model consisting of seven hypoxia-related gene signatures, and the model manifested high accuracy in predicting patients’ prognoses at 1, 3, and 5 years. Subsequent univariate and multivariate COX regression analyses eventually identified PSMB10, PSMD12, UBB, PSMA5, and PSMB6 as independent prognostic markers.

PSMA5, PSMB6, and PSMB10 are all subunits (α and β subunits) of the 20S proteasome core complex. This proteasome is a large multisubunit complex that is involved in protein degradation *via* the ubiquitin–proteasome pathway. Moreover, it is mainly associated with various biological processes, such as cell cycle, apoptosis, angiogenesis, cell adhesion, and transcription. The assembly of the eukaryotic 20S proteasome is thought to begin with the formation of the α-loop and requires the involvement of PSMA5 (30, 31). Previous studies have reported that PSMA5 mRNA expression levels are highly expressed in the serum of patients with sepsis presenting with hypoxemia but are lowly expressed in an *in vitro* hypoxia model (32). PSMB6 is associated with the progression of chronic hypoxic pulmonary hypertension and is involved in pulmonary vascular remodelling in hypoxia-induced rats (33). In addition, PSMB6 is upregulated in hypoxia models, lung cancer, and mesenchymal thyroid cancer (33–35). PSMB10 has also been shown to be a prognosis-related Hub gene in endometrial cancer (36). In the present study, PSMA5 and PSMB6 were found to be highly expressed in gliomas, while PSMB10 was found to be lowly expressed by integrating the TCGA and GTEx databases’ glioma sample analyses. PSMD12 was found in foetuses with neurodevelopmental disorders characteristic of autism and craniofacial anomalies, clubfoot, and syndactyly (37, 38). Disruption assays also support the biological importance of PSMD12 in proteasome function, especially during development and neurogenesis (39). In addition, PSMD12 expression is reportedly upregulated in glioma tissues compared with normal brain tissues and positively correlated with glioma grade. Zhang et al. (40) constructed a PSMD12-containing prognostic model for hypoxia in colorectal cancer and verified its high accuracy. UBB is a ubiquitin gene, a protein found in eukaryotic cells. The ubiquitin system helps regulate protein turnover. Ubiquitin attaches to the proteins that are to be degraded, effectively labelling them, and then the proteins are taken to a structure called the proteasome to form the ubiquitin–proteasome system (UPS). The UPS system can affect the survival of tumour cells by either promoting the interpretation of oncogenic proteins such as P53 or by blocking the degradation of oncogenic proteins (41). The components of the UPS system have become feasible targets for the development of potentially effective drugs against certain diseases, including some of these drugs that are already in clinical use or in the experimental phase. However, the UPS system is the primary pathway for intracellular protein degradation, thus hindering the development of protein degradation-based drugs, with only about 5% of Food and Drug Administration (FDA)-approved drugs currently targeting UPS system components (42, 43). Similarly, the five gene signatures in the prognostic prediction model constructed in this study are UPS system components.

Combining the above findings, we can identify the important roles of PSMB10, PSMD12, UBB, PSMA5, and PSMB6 in gliomas, thus providing new targets and ideas for tumour-targeted therapy. The present study has some limitations. Given that there is a dearth of research analysing the genes in tumours, the discovery of their mechanisms of action still needs improvement. Therefore, more biological experiments are needed to prove whether the conclusions reached can be verified *in vivo* or *in vitro*.

In summary, we constructed a prognostic model for glioma based on seven hypoxia-related genes and further identified five independent predictors of prognosis in glioma patients, thereby providing potential new targets for glioma gene-targeting therapy.

## Data Availability Statement

The original contributions presented in the study are included in the article/**Supplementary Material**. Further inquiries can be directed to the corresponding author.

## Author Contributions

All authors listed have made substantial, direct, and intellectual contribution to the work and approved it for publication.

## Funding

This study was supported by Ningbo public welfare science and technology program (No. 20181JCGY020333).

## Conflict of Interest

The authors declare that the research was conducted in the absence of any commercial or financial relationships that could be construed as a potential conflict of interest.

## Publisher’s Note

All claims expressed in this article are solely those of the authors and do not necessarily represent those of their affiliated organizations, or those of the publisher, the editors and the reviewers. Any product that may be evaluated in this article, or claim that may be made by its manufacturer, is not guaranteed or endorsed by the publisher.

## Supplementary Material

Supplementary Figure 1 Association of hypoxia-associated genes with characteristics and survival in different subgroups of patients.
