# Supplementary figures and images for: A Hypoxia-Associated Prognostic Gene Signature Risk Model and Prognosis Predictors in Gliomas
Source: Front Oncol. 2021 Nov 12;11:726794. doi: 10.3389/fonc.2021.726794 (PMC8632947; doi:10.3389/fonc.2021.726794)

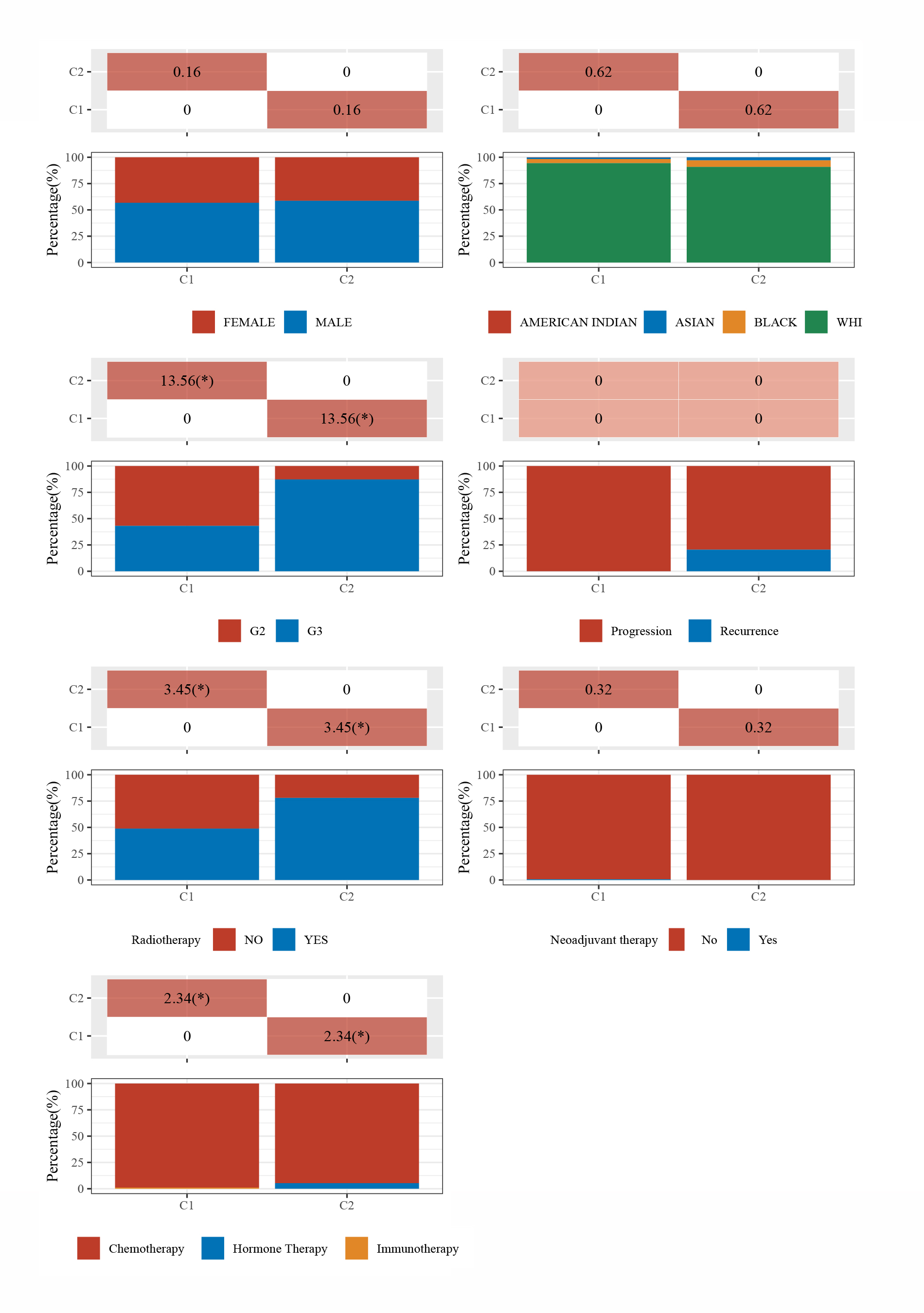

Supplement: Supplementary Figure 1 — Association of hypoxia-associated genes with characteristics and survival in different subgroups of patients. [file Image_1.jpeg]
